# Sex effect on the correlation of immunoglobulin G glycosylation with rheumatoid arthritis disease activity

**DOI:** 10.3906/biy-2005-7

**Published:** 2020-12-14

**Authors:** Altan ERCAN

**Affiliations:** 1 Department of Molecular Biology and Genetics, Faculty of Life and Natural Sciences, Abdullah Gül University, Kayseri Turkey

**Keywords:** IgG, glycosylation, sex, immune response, inflammation

## Abstract

Rheumatoid arthritis (RA) is a chronic autoimmune disease which affects females more than males with a presence of autoantibodies. Immunoglobulin G (IgG) produced by adaptive arm has 2 functional domains, Fc and Fab. The Fc domain binds Fc gamma receptors and C1q proteins of the innate arm. Therefore, the IgG Fc domain serves as a bridge between the innate and adaptive arms and is regulated by an evolutionarily conserved N-glycosylation with variable structures. These glycans are classified as agalactosylated G0, monogalactosylated G1, and digalactosylated G2, which are further modified by core-fucosylation (F) and bisecting N-acetylglucosamine (B) moieties such as G0F and G0FB. Interestingly, proinflammatory G0F is shown to be regulated by estrogen in vivo. Here, it is hypothesized that the regulation of G0F by estrogen contributes to sex dichotomy in RA by setting up the level of IgG-dependent inflammation and therefore, RA disease activity (Das28-CRP3). To investigate this hypothesis, IgG glycosylation was characterized in serum samples from active RA patients (n = 232) and healthy controls (n = 232) by serum N-glycan analysis using the high performance liquid chromatography. According to the results, the IgG Fc glycan phenotype originates predominantly from the structure of G0F, and both G0F and G0FB correlate with Das28-CRP3 in females, but not in males. In conclusion, IgG G0F-dependent inflammation differs in males and females, and these differences point to the differential regulation of inflammation by sex hormone estrogen via IgG glycosylation.

## 1. Introduction

Rheumatoid arthritis (RA) is a chronic autoimmune disease with unknown etiology affecting 0.5%–1.0% of the world population and more females than males with a ratio of 3 to 1 (Silman and Pearson, 2002). RA is characterized by the presence of autoantibodies such as rheumatoid factor (RF) and anticitrulline peptide (ACCP), the inflammation of the joints, and damage to cartilage and bone tissue in the joints (Young et al., 1979; Wozniczko-Orlowska and Milgrom, 1982; Hassfeld et al., 1989; Masson-Bessiere et al., 2001; Van Boekel et al., 2002; Vossenaar et al., 2004; Matsuo et al., 2006; Skriner et al., 2006; Verpoort et al., 2007; Hueber et al., 2009; Uysal et al., 2009; Smolen et al., 2016). Immunoglobulin G (IgG) is the second most abundant protein in the blood and a glycoprotein that provides the link between the 2 distinct arms of the immune system, the innate and the adaptive arms.

**Figure 1:**
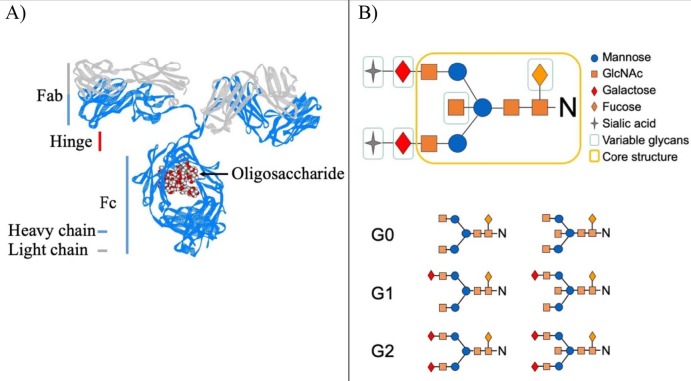
Structure of IgG and Fc glycans. (A) Shown above is the crystal structure of IgG. IgG consists of two heavy chain (blue), two light chain (gray) and oligosaccharide (red and white balls). The functional parts (Fc, Fab and hinge) of IgG formed by these heavy and light chains are shown with vertical lines suitable for their sizes. This figure is generated form crystal structure in PDB data bank file 1IGT using Viewer Lite software. (B) The fully (top) and partially (bottom) processed IgG Fc glycan structures and the glycan monomers forming this structure are shown (top-right corner).

IgG is produced by the complex interactions between T and B cells (Allen et al., 2007; Biram et al., 2019; De Silva and Klein, 2015; Victora and Nussenzweig, 2012; Suan et al., 2017). IgG consists of 2 heavy and 2 light chains resulting in 2 functional domains, Fc and Fab connected by the flexible hinge region (Figure 1) (Harris et al., 1997; Schroeder and Cavacini, 2010; Jefferis, 2012; Vidarsson et al., 2014; Jay et al., 2018). While the Fab domain binds to the antigen with high affinity, the Fc domain initiates the IgG-dependent cell and complement-dependent cytotoxic pathways by stimulating the innate arm of the immune system (Pincetic et al., 2014; Vidarsson et al., 2014; Bournazos and Ravetch, 2017; Peschke et al., 2017; Quast et al., 2017). The IgG Fc domain contains an evolutionarily conserved N-glycan attached to Asparagine 297 residue (Figure 1a) (Taylor et al., 2009; Bournazos and Ravetch, 2017; Bournazos, 2019). This N-glycan is a complex two-antenna structure which begins with N-acetylglucosamine (GlcNAc) and is elaborated by the addition of GlcNAc, mannose, GlcNAc, and galactose capped with sialic acid (Figure 1b) (Watson et al., 1999; Jefferis, 2012).

N-glycan in the IgG Fc domain is heterogeneous, which results from the differential activity of synthetic glycosyltransferases during its production, and has been historically classified with respect to the galactose content as no galactose (G0), one galactose (G1), and 2 galactose (G2) (Figure 1b) (Axford, 1999). This N-glycan contains at least 36 different structures when characterized using liquid chromatographic techniques (Royle et al., 2008). In healthy people, these glycan structures G0 and G2 vary between 20% and 40%, while G1 is around 40% (Holland et al., 2006).

IgG N-glycan is different from the other known glycans. In general, glycans decorate the outer surface of the proteins, but N-glycan in the IgG Fc fragment is sequestered in a aspace formed by 2 heavy chains of IgG (Figure 1a) (Harris et al., 1997; Gagneux and Varki, 1999; Raju and Lang, 2014). In this space, N-glycans modify the outer surface of the IgG Fc domain and thus affect the binding affinity of the IgG Fc fragment toward the Fc gamma receptors (Lund et al., 1990; Malhotra et al., 1995; Anthony et al., 2011; Ahmed et al., 2014; Subedi et al., 2014; Nandakumar et al., 2018). Therefore, the variation on the IgG Fc glycan alters IgG-dependent immune response such as antibody-dependent cell cytotoxicity (Lund et al., 1990; Malhotra et al., 1995; Anthony et al., 2011; Ahmed et al., 2014; Subedi et al., 2014; Nandakumar et al., 2018). In addition, less processed IgG Fc N-glycan structures, which do not contain galactose and end with GlcNAc (G0), activate the mannose-dependent lectin pathway, resulting in complement-dependent cytotoxicity (Malhotra et al., 1995). In short, the data in the literature prove that different IgG Fc N-glycan structures lead to different physiological outcomes (Malhotra et al., 1995; Kaneko et al., 2006; Anthony and Ravetch, 2010; Ferrara et al., 2011; Golay et al., 2013; Nandakumar, 2018; Nandakumar et al., 2018). It is even suggested that fully processed IgG Fc glycan ending up with sialic acid is antiinflammatory (Kaneko et al., 2006; Anthony et al., 2011; Ahmed et al., 2014).

This different glycan structure results from the differential regulation of the glycosyltransferase and glycosidase enzyme activities in the endoplasmic reticulum-golgi (ER-Golgi) during the synthesis and secretion of IgG in B cells. With a limited data on the literature, it is also possible that IgG N-glycans in circulating blood can be processed by glycosyltransferases (Pagan et al., 2018). The data in the literature point to health status and hormone levels as the source of these different glycan structures, though not in a detailed way (Van de Geijn et al., 2009; Wang et al., 2011; Bondt et al., 2013; Ercan et al., 2017). The most striking one among the studies about hormonal regulation of IgG glycosylation is the control of IgG galactosylation by estrogen in both men and women (Ercan et al., 2017).

RA is an autoimmune disease that begins with the wrongful activation of the adaptive arm of the immune system for unknown reasons, which results in the production of antibodies specific for autoantigens. In RA, it was demonstrated that ACCP antibodies are present in serum at least 4.1 years before the diagnosis of RA (Holers, 2007). Studies with RA animal models have undoubtedly proven the importance of autoantibodies in the onset and progression of RA, especially in the antibody-dependent K/BxN mouse model (Matsumoto et al., 2002; Monach et al., 2004; Monach et al., 2008). For example, the phenotype of RA disease can be transferred from a K/BxN mouse with active RA to a naive C57BL/6 mouse by transferring the IgG purified from the serum of the K/BxN mouse with active RA ( Korganow et al., 1999; Maccioni et al., 2002; Matsumoto et al., 2002; Ditzel, 2004; Monach et al., 2004). In addition, the transfer of immune cells without B cells from an arthritic DBA/1 mouse fails to initiate an RA-like phenotype in anaive B and T cell-deficient SCID mouse, providing evidence for the importance of the adaptive arm of the immune system and the production of IgG without a doubt (Tanaka-Watanabe et al., 2009). This is further supported by autoantibodies such as anti-CCP and RF in humans, which successfully serve as a biomarker for the diagnosis of RA, and this points to the importance of IgG in disease mechanism (Atzeni et al., 2017).

IgG Fc glycosylation determines the severity of IgG-dependent immune response which strongly correlates with the level of G0t, a combination of agalactosylated G0F and G0FB glycan structures. As the level of G0t increases, the cumulative score of disease activity (Das28-CRP3) increases (Malhotra et al., 1995; Kaneko et al., 2006; Arnold et al., 2006; Arnold et al., 2007; Nandakumar et al., 2007; Anthony et al., 2008; Anthony and Ravetch, 2010). Interestingly, a group of researchers including the author of the present study proved that the galactosylation of IgG is controlled by estrogen (Ercan et al., 2017). In addition, a sex-dependent correlation was noted between G0t and Das28-CRP3, which is present in females but not in males (Ercan et al., 2010). Here, it is hypothesized that the correlation between Das28-CRP3 and G0t originates from the G0F glycan structure and this is sex-dependent. To investigate this hypothesis, the correlation of Das28-CRP3 with G0F and G0FB glycan structures is examined in detail focusing on sex difference.

## 2. Materials and methods

### 2.1. Study population

Serum samples for the IgG glycan analysis were obtained from the patient sample bank of the Brigham Rheumatoid Arthritis Sequential Study (BRASS). The BRASS cohort characteristics are described in detail in the study by Ercan et al. (2010). Briefly, the serum samples used in this study were taken from active RA patients treated at Brigham and Women’s Hospital (Table). After obtaining the blood samples, the serum samples were generated and kept frozen at −80 °C until it was used. The effects of freeze and thaw cycles on the IgG glycan were analyzed and no effects were observed. Age- and sex-matched serum samples from anonymous healthy people were used as controls for the BRASS RA cohort. The cohort properties of these examples are summarized in Table. This study was approved by the Brigham and Women’s Hospital ethics committee.

**Table .** Characteristics of the cohorts.

	BRASS RA patients (n =232)	Healthy controls (n = 232)
Age (years) average ± SD	57.9 ± 12.8	52.6 ± 14.1
Female (%)	119 (51.3)	119 (51.3)
Das28-CRP3 average ± SD	4.145 ± 1.725	—
G0t average ± SD	1.36 ± 0.41	1.01 ± 0.23
G0F average ± SD	0.85 ± 0.34	0.61 ± 0.19
G0F average ± SD	0.50 ± 0.13	0.40 ± 0.10

### 2.2. Determination of glycan peaks belonging to IgG in serum sample

To identify the glycan structures originating from IgG, 3 samples were generated and characterized for each serum sample (the original serum sample, the IgG-depleted serum sample, and the purified IgG). For the purification of IgG from the serum sample, Protein G-Sepharose beads were used because it binds to IgG with very high affinity and specificity. First, the Protein G beads were equilibrated with 100 mM Tris-HCl pH 7.5 on the benchtop. Then, the serum sample was applied to the column. The material that passed through the column without interacting is the IgG-depleted serum sample. Then, the column was washed with 100 mM Tris-HCl pH 7.5 buffer 5 times the volume of the column to get rid of the residual unbound materials. The IgG bond to Protein G beads was eluted with 100 mM Acetic acid at pH 2.5. As soon as the sample was eluted from the column, the pH of the sample was adjusted to pH 7.5 by the addition of 1.5 M Tris-HCl at pH 10. This sample was a purified IgG. The serum, IgG-depleted serum, and protein G purified IgG samples were analyzed by the serum N-glycan analysis using high performance liquid chromatography (HPLC) (Royle et al., 2008; Ercan et al., 2010). According to their profiles, the N-glycan structures of IgG were determined.

### 2.3. Serum IgG N-glycan analysis

The N-glycan analysis of the serum samples from RA patients and healthy controls were performed by the serum HPLC-based N-glycan analysis (Royle et al., 2008; Ercan et al., 2010). The serum samples (5 µL) were transferred to each well of a polypropylene flat-bottomed 96-well microplate. The serum samples were mixed with 2 µL of sample buffer containing 1 mL of 10% SDS, 0.625 mL of 0.5 M Tris-HCl, pH 6.6, 3.4 mL of water, 2 µL of water, and 1 µL of 0.5 M dithiothreitol. Following the incubation of the samples at 65 °C for 15 min to reduce proteins, the samples were alkylated by the addition of 1 µL of 100 mM iodoacetamide followed by incubation at room temperature for 30 min in dark. The reduced and alkylated samples were immobilized in 100 µL of 10% SDS-PAGE gel. The gel pieces containing immobilized samples were transferred into 2 mL, 0.45 μm hydrophilic PVDF, a filter-bottom 96-well microplate. Then, the samples were washed with 1 mL of 20 mM NaHCO3, pH 7.2 followed by 1 mL of acetonitrile. This washing step was repeated 5 times. The samples were dried using vacuum centrifuge. Then, the N-glycans in the immobilized samples were cut off using the PNGase F enzyme according to the procedure suggested by the producer. The N-glycans detached from proteins were collected in a 2 mL square-tapered polypropylene 96-well plate by washing the gel pieces 3 times with 200 mL of water followed by washing 3 times with 200 mL of acetonitrile. The collected samples were dried and treated with 20 µL of 1% acetic acid at room temperature for 40 min to open the reducing end of the glycans. These reducing ends that were opened were labeled with 2-aminobenzamide (2AB) using a LudgerTag 2AB labeling kit. The 2AB-labeled N-glycans were transferred onto a Whatman 3MM chromatography paper and placed into a 96-well plate. Following washing 5 times with 2 mL of acetonitrile, the labelled and cleaned glycans were eluted with 0.9 mL of water twice. The samples were then dried, resuspended in water, and separated on a TSKgel Amide-80 column (4.6 mm in internal diameter and 25.0 cm in length) attached to HPLC with the gradient of ammonium format (35% **→** 47%): acetonitrile (65% **→** 53%) in 60 min (Royle et al., 2008). The quantitative data for the G0F, G0FB, and G1t glycans were obtained from the areas under each glycan peak.

### 2.4. Statistical analysis

The differences between the patient and control groups were analyzed using the Student’s t-test. The normal distribution of the data groups was analyzed using the Shapiro–Wilk test. According to the results, all the groups used here had a normal distribution. Therefore, the correlations of the G0t, G0F, and G0FB glycans with Das28-CRP3 disease activity were analyzed by the Spearman’s rho value.

## 3. Results

### 3.1. The use of GOF and G0FB glycan structures by IgG in serum

**Figure 2:**
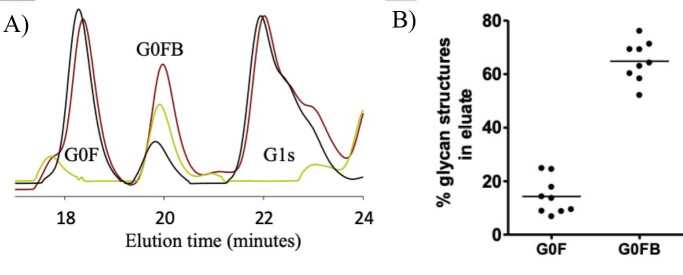
Characterization of IgG N-glycans in serum samples. (A) Shown are N-glycan profiles of serum (brown), IgG-depleted (yellow) and purified IgG (black) samples, and peaks of G0F, G0FB and G1t glycan structures to which the peaks belong. (B) The areas under the G0F and G0FB peaks are calculated from serum and IgG isolated serum samples and summarized in the graph above.

Here, the glycan profiles obtained from 3 samples are examined in detail. According to the comparison of the protein G-purified IgG glycan profile with serum alone and the IgG-depleted serum N-glycan profiles, the G0 structures in the serum N-glycan profile mainly originate from IgG, while the G2 glycan structures has a significant contribution from other proteins in the serum (Figure 2a). In the detailed examination of the G0 glycan structures, the G0F and G0FB glycan structures that make up the G0t glycans had contributions from different glycoproteins in the serum (Figure 2b). When the N-glycan profile of the serum samples (n = 9) was compared with that of the matched IgG-depleted serum sample, the G0F glycan structure (14.3 ± 6.7%) and the G0FB structure (64.48 ± 7.4%) originated from other glycoproteins in the serum. After determining the contributions from serum glycoproteins to the IgG G0F and G0FB glycans, the sex dependency of the correlation of the G0F and G0FB glycan structures with the collective disease actives score Das28-CRP3 was examined.

### 3.2. Contributions of G0F and G0FB glycan structures to G0t phenotype in RA

**Figure 3:**
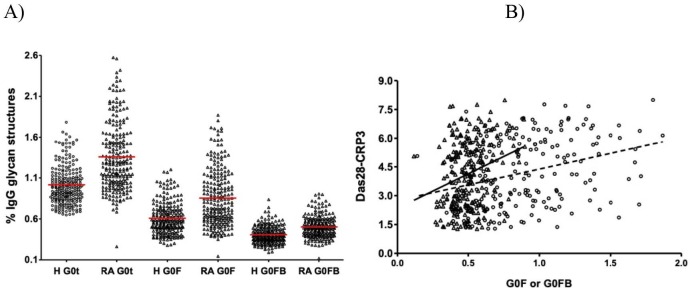
Contributions of G0F and G0FB glycan structures to the correlation between G0t phenotype and Das28-CRP3. (A) The % values of G0t, G0F and G0FB glycansin RA patients (n = 232) were compared that of healthy human (H) samples (n = 232) using Student’s t-test, and they are all statistically different. (B) The correlation of G0F (circle, dashed line, and n = 226) and G0FB (triangle, straight line, and n = 226) glycan structures with Das28-CRP3 was presented and assessed using the Spearman rank coefficient method.

The contribution of the G0F and G0FB glycan structures to the G0t phenotype in RA was examined. In healthy controls (n = 232), G0t (1.01 ± 0.23) was composed of 0.61 ± 0.19 G0F and 0.40 ± 10 G0FB structures (Figure 3a). In RA patients (n = 232), G0t (1.36 ± 0.41) was composed of 0.85 ± 0.34 G0F and 0.50 ± 0.13G0FB structures. Although the contribution of G0F (~61%) and G0FB (~39%) structures to G0t in RA patients and healthy controls did not differ in percentage, the quantities of G0F (average ± SD, 0.85 ± 0.34 vs. 0.61 ± 0.19; P < 0.0001) and G0FB (average ± SD, 0.50 ± 0.13 vs. 0.40 ± 0.10; P < 0.0001) structures were statistically different (Figure 3a). These data show that the G0F and G0FB glycan structures together contribute to the G0t phenotype in RA disease compared to healthy controls and both the G0F and G0FB glycan structures are different in RA patients compared to healthy controls (P < 0.0001) (Figure 3a).

### 3.3. Correlation of G0F and G0FB N-glycan structures with Das28-CRP3 is sex-dependent

**Figure 4:**
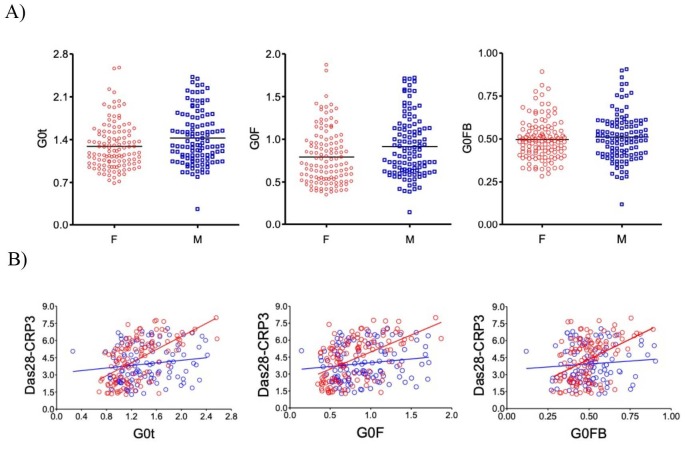
Sex dependent differences of the IgG G0t, G0F and G0FB glycan structures and the correlations with Das28-CRP3. (A) The distribution of G0t (left), G0F (middle) and G0FB (right) glycan structures in women (F, red, n = 119) and men (M, blue, n = 107) are shown above. The presence of differences of G0t, G0F and G0FB between M and F was evaluated using Student's t-test. (B) Correlation of these glycan structures with Das28-CRP3 in women (red circles and line) and men (blue circles and line) is shown.

When the known correlation of the G0t glycan structures with Das28-CRP3 is examined for G0F and G0FB, both glycan structures have different correlations with Das28-CRP3. The correlation of the G0F structure (Spearman ρ = 0.34, P < 0.0001) is higher than that of the G0FB structure (Spearman ρ = 0.24, P = 0.0001) (Figure 3b). When this data is stratified by sex, the G0F glycan structure (P = 0.0058) is statistically different while the G0FB glycan structure (P = 0.38) is not statistically different (Figure 4a). Further examination for the correlations of the G0t, G0F, and G0FB glycan structures with Das28-CRP3 yields the values for G0t (Spearman’s ρ = 0.60, P < 0.0001), G0F (Spearman’s ρ = 0.60, P < 0.0001), and G0FB (Spearman’s ρ = 0.53, P <0.0001). When the sex dependence of these correlations is investigated, the structures of G0t (Spearman’s ρ = 0.60, P < 0.0001), G0F (Spearman’s ρ = 0.53, P < 0.0001), and G0FB (Spearman’s ρ = 0.39, P < 0.0001) have strong correlations in female RA patients while G0t (Spearman’s ρ = 0.16, P = 0.1032), G0F (Spearman’s ρ = 0.180, P = 0.1327), and G0FB (Spearman’s ρ = 0.080, P = 0.4146) do not correlate in male RA patients (Figure 4b). Therefore, the correlation of G0t with Das28-CRP3 originates from both the G0F and G0FB glycan structures, but this correlation is sex-dependent.

### 3.4. The effect of menopause on the correlation of G0t, G0F, and G0FB with Das28-CRP3

**Figure 5:**
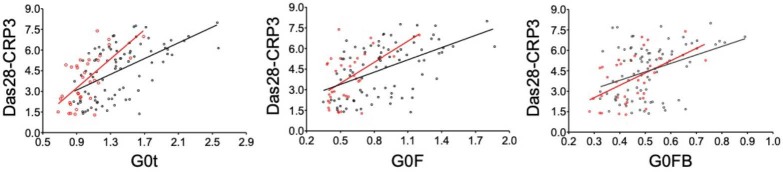
The effect of menopause on the correlation of IgG G0t, G0F and G0FB glycan structures with Das28-CRP3. In the graphs above, the correlations of G0t (left), G0F (middle) and G0FB (right) glycan structures with Das28-CRP3 are evaluated using the Spearman rank coefficient method and shown as premenopause (red circle and line, n = 34) and postmenopause (black circle and line, n = 85).

The effect of menopause on the correlation of the G0t, G0F, and G0FB glycan structures with Das28-CRP3 is investigated. For menopause, the age of 50 is accepted as a critical age and female RA patients aged 50 years and below are classified as premenopause and those aged above 50 years as postmenopause. According to the results, the G0t glycan structure has correlations with Das28-CRP3 in the premenopause (Spearman’s ρ = 0.63, P < 0.0001) and postmenopause (Spearman’s ρ = 0.57, P < 0.0001) groups (Figure 5). The G0F glycan structure correlates with Das28-CRP3 in premenopause (Spearman’s ρ = 0.43, P = 0.011) and postmenopause (Spearman’s ρ = 0.51, P < 0.0001) (Figure 5). Likewise, the G0FB glycan structure correlates with Das28-CRP3 in the premenopause (Spearman’s ρ = 0.46, P = 0.0061) and postmenopause (Spearman’s ρ = 0.32, P = 0.0031) groups (Figure 5). Although all the IgG glycan structures examined here differ in their level of correlations with Das28-CRP3, they all have statistically significant correlations.

## 4. Discussion

The immune system differs in men and women, and the reason for this is not fully understood to date (Klein, 2012a; Klein, 2012b; Ngo et al., 2014; Rainville et al., 2018; Dolsen et al., 2019). A good example for this is that RA disease affects females more than males with a ratio of 3/1. The studies with pregnant RA patients, whose estrogen level peaks, point to the relationship between the G0t glycan structures and the Das28-CRP disease activity. In this patient group, as the level of the G0t glycan structure decreases, the Das28-CRP3 score decreases (Reiding et al., 2017; Bondt et al., 2018). Recent research aligned with this observation proves that the sex hormone estrogen regulates the distribution of IgG glycan structures in vivo (Ercan et al., 2017). Because of these observations, a detailed investigation was performed on the relationship between different proinflammatory G0F and G0FB glycan structures and Das28-CRP3. The data presented here show that IgG-dependent inflammation in females and males contributes to the sex dichotomy in the immune system by utilizing different distribution of the IgG Fc glycan structures in males and females.

The glycan analysis was performed using the HPLC based serum N-glycan analysis (Royle et al., 2008). In the serum, more than 50% of the proteins in the human body contain glycosylation (Apweiler et al., 1999). In parallel, the serum N-glycan profile is also a complex glycan mixture profile, and different proteins may contain the same glycan structures as in IgG and fibrinogen (Ercan et al., 2010; Adamczyk et al., 2013; Adamczyk et al., 2017). IgG and fibrinogen glycoproteins contribute to the serum N-glycan profile with two-antenna complex N-glycan structures. Although the fibrinogen N-glycan profile is more processed and predominantly capped with sialic acid structure compared to the IgG N-glycan profile, IgG has more diverse glycan structures—at least 36 glycan structures—which are more evenly distributed ( Royle et al., 2006; Adamczyk et al., 2013). Thus, to examine the contribution of the IgG N-glycan structures to the serum N-glycan profile, 3 samples were generated as the serum, IgG-depleted serum, and protein G-purified IgG, which were characterized using the HPLC-based serum N-glycan analysis method (Royle et al., 2008). When the IgG-isolated serum N-glycan and serum N-glycan profiles were compared, 86% of the G0F glycan structure originated from IgG while 36% of the G0FB structure originated from IgG. Therefore, it is reasonable to consider the data obtained for the G0F glycan structure using serum N-glycan profile heavily belongs to IgG in the serum.

The G0F and G0FB glycan structures contribute to the G0t phenotype in RA patients (Figure 3a), and both the G0F and G0FB glycan structures have correlations with Das28-CRP3 (Figure 3b). However, when RA patients are stratified by sex, the G0F glycan structure is expressed differently in males and females while the G0FB glycan structure is not (Figure 4a). In addition, when the correlations of the G0F and G0FB glycan structures with Das28-CRP3 are examined, there is a tight correlation in females but not in males. Therefore, these results indicate a new axis of sex-dependent regulation of inflammation via the G0F glycan structure.

To understand the effect of estrogen on the correlations of the G0F and G0FB glycan structures with Das28-CRP3 in more detail, the effect of sex and menopause is investigated. The G0F glycan structure has a strong correlation with Das28-CRP3 in females but not in males, indicating that IgG glycosylation, more specifically the G0F glycan structure, contributes to the sex dichotomy in RA and IgG-dependent inflammation. When the effect of menopause, which is an important turning point in hormonal levels in the life of women, is investigated, the G0F and G0FB glycan structures surprisingly have correlations with Das28-CRP3 in both premenopause and postmenopause. This can be due to the estrogen supplement taken by postmenopausal females (Engdahl et al., 2018). The results presented here and the data in the literature grant a more detailed investigation to dissect the effect of menopause on the association of G0F and G0FB with Das28-CRP3 (Ercan et al., 2017; Reiding et al., 2017; Bondt et al., 2018).

In general, the sex hormone estrogen modulates IgG glycosylation and therefore the communication between the adaptive and innate arms of the immune system (Ercan et al., 2017). IgG plays an important role in the activation of the innate system, the other arm of the immune system, through interaction with FcgR and the complement fragment C1q molecules (Nicholson, 2016). The importance of IgG binding to FcgR and C1q molecules has been proven without doubt in RA mouse models. In RA mouse models, the transfer of anti-CCP and anti-GPI pathogenic autoantibodies purified from sick mice to healthy mice has been shown to cause inflammation and related destruction of bone and cartilage in small joints, and this proinflammatory effect of IgG disappears with the removal of IgG Fc glycan (Allhorn et al., 2010; Lood et al., 2012). Therefore, the proinflammatory effect of IgG depends on the N-glycan structure in the IgG Fc fragment and is critical for this process. Based on the data presented here about the differential correlation of the IgG glycan structures with Das28-CRP3 in women and men, the sex-dependent regulation of the proinflammatory property of IgG depends on IgG Fc glycosylation, which is regulated by hormones. In conclusion, the communication between innate and adaptive immune systems through the IgG Fc glycan structures is differentially regulated in men and women via IgG glycosylation, more specifically the G0F glycan structure.
